# Retrospective analysis of 217 fatal intoxication autopsy cases from 2009 to 2021: temporal trends in fatal intoxication at Tongji center for medicolegal expertise, Hubei, China

**DOI:** 10.3389/fpubh.2023.1137649

**Published:** 2023-04-17

**Authors:** Lin Lihua, Wang Yuning, He Henghui, Liu Xiang, Jiang Min, Li Zehao, Li Lianjie, Liu Qian

**Affiliations:** Department of Forensic Medicine, Tongji Medical College of Huazhong University of Science and Technology, Wuhan, China

**Keywords:** poisoning, forensic, toxicology, temporal trends, autopsy data, retrospective analysis

## Abstract

This retrospective analysis of fatal intoxication case autopsies was performed at Tongji Center for Medicolegal Expertise in Hubei (TCMEH) from 2009 to 2021 to obtain up-to-date information on intoxication cases. The objective was to describe important data about evolving patterns in intoxication occurrences, enhance public safety policies, and assist forensic examiners and police in more efficient handling of such cases. Analyses based on sex, age, topical exposure routes, toxic agents, and mode of death were performed using 217 records of intoxication cases collected from TCMEH as a sample, and the results were compared with reports previously published (from 1999 to 2008) from this institution. Deaths from intoxications occurred at a higher rate in males than in females and were most common among individuals aged 30–39 years. The most frequent method of exposure was oral ingestion. The causative agents of deadly intoxications have changed when compared to the data from the previous 10 years. For instance, deaths from amphetamine overdoses are becoming more prevalent gradually, whereas deaths due to carbon monoxide and rodenticide intoxication have declined dramatically. In 72 cases, pesticides continued to be the most frequent intoxication cause. A total of 60.4% of the deaths were accidental exposure. Men died from accidents at a higher rate than women, although women were more likely to commit suicide. Particular focus is needed on the use of succinylcholine, cyanide, and paraquat in homicides.

## 1. Introduction

Intoxication is a global public health concern. In 2015, accidental intoxications caused 86,400 fatalities worldwide at a rate of 1.2 per 100,000 people (1). More than 90% of the intoxication-related deaths occur in lower middle-income countries (2). China is an emerging agriculturally-based nation, therefore, pesticide intoxication is one of the most prevalent forms of intoxication in China. However, the tendency toward intoxication has changed considerably due to the rapid urbanization and regulatory prohibitions on the use of particular toxicants (3). Therefore, it is anticipated that characteristics of intoxications and hazardous compounds linked to the deaths may alter.

The tremendous economic growth and changing lifestyles in China over the last few decades may have impacted the rising intoxication events. Our data may provide evidence regarding the outcome of interventions and suggest additional decisions to address intoxications. The retrospective data reported in this study could be a valuable resource for forensic pathologists and police officers dealing with intoxication cases. This is because there are currently no official statistics on autopsy data of intoxication deaths in China. It can also be a reference for identifying temporal trends in intoxication events and creating public health intervention strategies.

Our research group previously reported intoxication deaths in the Tongji Center for Medicolegal Expertise in Hubei (TCMEH) during the years 1999 through 2008 (4), which were contrasted with the intoxication cases recorded in the most recent 13 years (2009–2021).

## 2. Materials and methods

### 2.1. Study setting and case sources

TCMEH is a forensic institution affiliated with the Department of Forensic Medicine, Tongji Medical College, Huazhong University of Science and Technology, in Wuhan, Hubei. The institution accepts cases for investigation from Hubei and surrounding provinces, such as Henan, Hunan, Jiangxi, and Fujian. We retrospectively examined 4,753 autopsy documents recorded between January 2009 and December 2021 in TCMEH. Of the total 4,753 cases, we comprehensively evaluated the autopsy records, histopathology, toxicology test reports, case information, and scene evidence of the deceased and identified 217 cases with intoxication as the primary cause of death as the subjects of this retrospective analysis. Overall, 217 cases of fatal intoxications were included for further analyses after cases with conflicting causes of death or insufficient information were excluded. The deceased's kin completed written informed consent documentation. Data analyzed in this study were obtained from TCMEH with the approval of the Tongji Medical College Ethics Committee at Huazhong University of Science and Technology.

### 2.2. Toxicological analysis

For all 217 cases, toxicological analyses were performed either at our toxicological laboratory or the toxicological laboratories of the national, provincial, and local public security agencies using methods such as GC-MS/MS, LC-MS/MS, HS-GC, gold immunochromatographic assay, and UV-visible spectrophotometry, etc. To determine the potential existence of various toxicants and exposure pathways, samples of urine, blood (heart blood or peripheral blood), liver, kidney, stomach wall, and stomach contents were obtained. Specific biological materials of a few unique cases were examined. For instance, when skin contact intoxication was suspected, the local skin was tested for toxicity. Using these samples for toxicological testing is in line with the standards of the People's Republic of China public safety industry. The toxicological analysis can provide a good evidence, but the cause of death needs to be determined in conjunction with the autopsy report and the circumstances of the case. The blood concentration standards utilized for diagnosing fatal intoxications were in accordance with the pertinent national/industrial standards or based on the lethal dose facts published in national textbooks (5, 6). Depending on the nature of the toxicant or epidemiological characteristics of the case, nine different toxicants were categorized as follows: pesticides (rodenticides, insecticides, and herbicides); prescription medications; illicit drugs (narcotic drugs and addiction-inducing psychotropic substances); alcohol; toxic plants and animals; metal salts; combined intoxication; other compounds (e.g., nitrite, succinylcholine, and cyanide); and unidentified toxicants. Because of technological limitations, some early toxicant cases lacked qualitative data.

### 2.3. Causes and manner of death assessment

The likelihood of mechanical asphyxiation, mechanical trauma, or sickness was ruled out during autopsy, pathological investigation, and toxicity study. To determine that intoxication was the primary cause of death in each case, two forensic medical specialists thoroughly examined the data, including the briefing on the cases, clinical histories, autopsy records, and toxicology findings. A third forensic examiner then reviewed the final report before it was released. In China, the police, not the medical examiner, determine how an individual dies. The medical examiner's cause of death, case investigation, autopsy reports, and results of the toxicological study were all considered by the police when determining the manner of death.

### 2.4. Statistical analysis

Microsoft Excel 2016 was used to organize and summarize the data and generate the figures. IBM SPSS Statistics for Windows, version 27.0 (IBM Corp., Armonk, NY, USA) was used to describe the data, and the mean ± standard deviation are presented. To evaluate differences, χ^2^ test (*P*-value < 0.05) was performed.

## 3. Results

### 3.1. Incidence and trends

A total of 4,753 death cases, including 217 wherein intoxication was the principal cause of death, were accepted at TCMEH between 2009 and 2021. The yearly number of deaths due to intoxication ranged from 4 to 30 (average, 17), with the highest (6.4%) and lowest (1.9%) percentage of intoxication fatalities in 2013 and 2019, respectively. Compared to 1999–2008 (which reported 218 intoxication deaths out of 2416 deaths) (4), the mortality rate from forensic autopsy intoxications in this study fell by 4.4%. Table 1 shows the total number of autopsy cases and fatal intoxication cases caused by various toxicants each year.

**Table 1:** The different intoxication agents and the number of poisoning deaths per year from 2009 to 2021.

**Toxic agents category**	**2009**	**2010**	**2011**	**2012**	**2013**	**2014**	**2015**	**2016**	**2017**	**2018**	**2019**	**2020**	**2021**	**Total**
Rodenticide	0	2	1	3	6	1	1	5	0	0	1	0	0	20
Insecticides	8	5	3	4	5	6	9	2	1	1	1	2	0	47
Herbicides	0	1	0	0	0	0	0	1	0	2	1	0	0	5
Drugs	0	5	1	0	3	4	4	2	1	0	0	0	0	20
CO	0	1	3	0	5	1	2	0	0	2	1	0	3	18
Prescription medicine	0	0	2	0	0	1	1	0	2	0	0	1	1	8
Alcohols	4	3	6	5	5	7	5	2	2	2	0	0	1	42
toxic animals and plants	0	1	2	2	2	1	2	1	5	0	0	0	0	16
Other compounds	1	1	4	0	4	3	3	2	0	0	1	0	1	20
Metal (salts)	1	1	1	0	0	0	0	1	0	0	0	0	2	6
Combined intoxication	0	1	0	3	0	2	1	3	0	0	0	1	0	11
Unknown	1	0	0	0	0	1	1	1	0	0	0	0	0	4
Total (intoxication)	15	21	23	17	30	27	29	20	11	7	5	4	8	217
Total (autopsy cases)	341	380	382	400	470	498	545	406	338	354	270	140	229	4,753
Percentages (%)	4.4	5.5	6.0	4.3	6.4	5.4	5.3	4.9	3.3	2.0	1.9	2.9	3.5	4.6

### 3.2. Sex and age distribution

**Figure 1:**
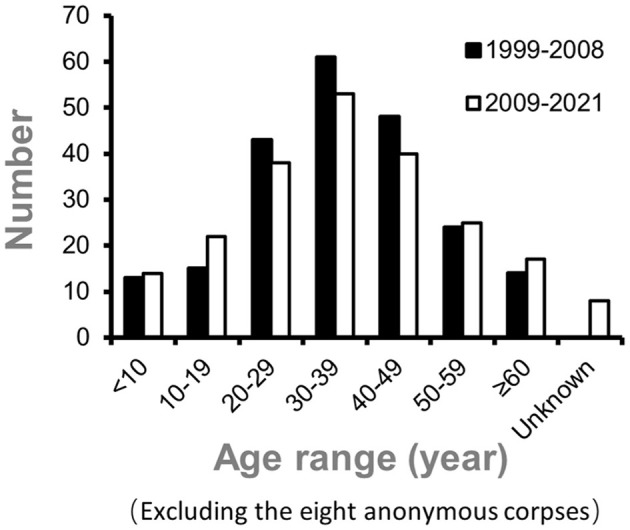
Age distribution of intoxication deaths between 2009–2021 and 1999–2008 periods at the Tongji Center for Medicolegal Expertise in Hubei, China (4).

**Figure 2:**
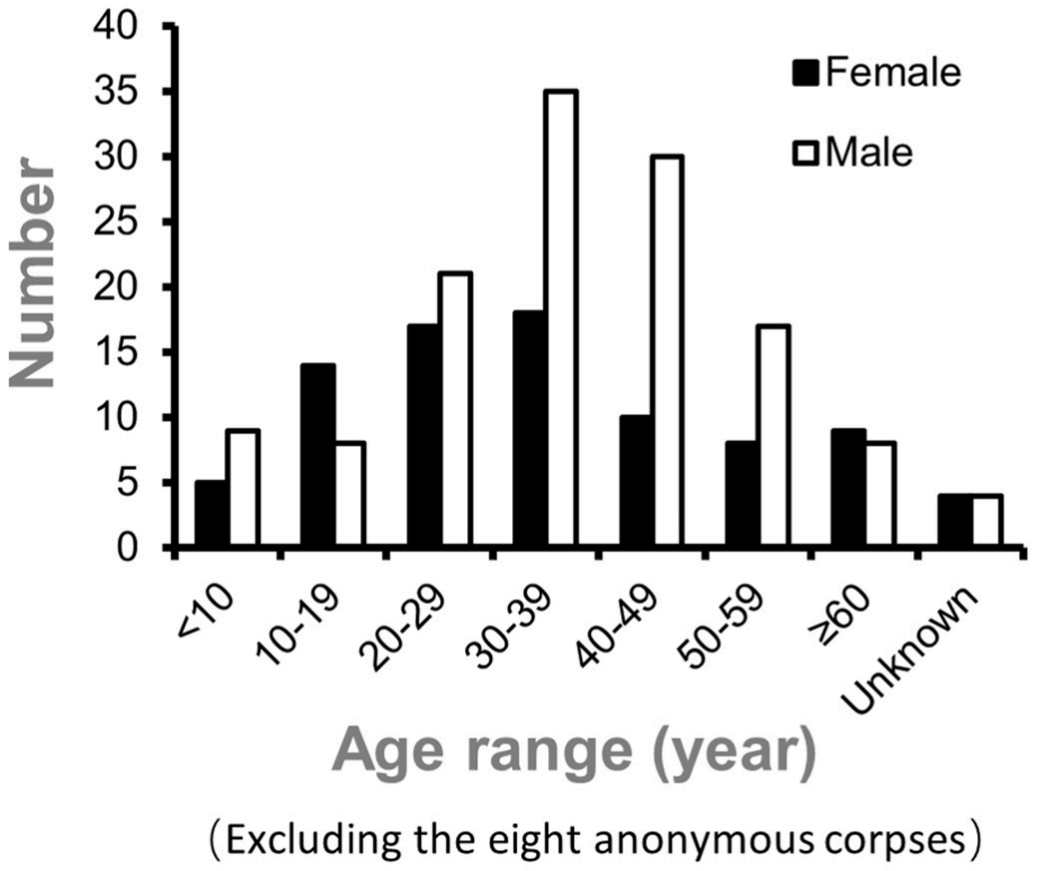
Sex and age distribution of fatal intoxication cases in 1999 to 2021.

Overall, 132 males (60.8%) and 85 females (39.2%) died from intoxications. The median age of the deceased was 36 years (36.0 ± 16.7; range, 7 months to 75 years; excluding the eight anonymous corpses). Patients' average ages were 37 ± 16.1 years for men and 36 ± 18.1 years for women. Figure 1 displays the age distribution of intoxication-related fatalities between 2009–2021 and 1999–2008. During the period 2009 to 2021, the age group for which the most fatal intoxication was identified included individuals between 30 and 39 years (24.4% of cases), followed by those aged between 40 and 49 years (18.4% of cases). Figure 2 displays the age and sex distribution of fatal intoxication cases from 1999 to 2021.

### 3.3. Routes of exposure

Routes of exposure in both this and previous studies (4) are listed in Table 2, indicating oral ingestion as the most common exposure route (66.4%), followed by inhalation (21.7%) and injection (5.1%). There were no significant differences between the 1999–2008 and 2009–2021 data.

**Table 2:** Distribution of exposure routes over the two periods (1999–2008 and 2009–2021) (4).

**Route of exposure**	**2009–2021**		**1998–2008**		χ^**2**^**-test**	
	***n***	**%**	***n***	**%**	χ^2^	***p*** **-value**
Oral ingestion	144	66.4	142	65.1	0.072	0.788
Inhalation	47	21.7	43	19.7	0.248	0.619
Injection	11	5.1	18	8.3	1.776	0.183
Dermal	5	2.3	5	2.3	–	1.000
Bite and Sting	2	0.1	4	1.8	0.164	0.685
Combined	7	3.2	4	1.8	0.854	0.356
Unknown	1	0.1	2	0.9	–	1.000

χ^2^, Chi-squared; showing no significant *p*-values.

### 3.4. Toxic agents

This study grouped all toxicants into nine classes and further categorized them into various subclasses. Compared to the previous report period (4), Table 3 shows the number and percentage of intoxication cases according to these classes and subclasses. Rodenticides, insecticides, and herbicides are types of pesticide. Methanol and ethanol are examples of alcohol. Amphetamines, heroin, and morphine are classified as illicit drugs. Combined intoxication refers to exposure to two or more types of toxicants. Other compounds included succinylcholine, nitrite, and cyanide. We classified arsenide and barium chloride as metal salts. In this study, pesticides were the main cause of intoxication mortality (33.2% of deaths), with organic phosphorus as the main cause (54.2%). The highest number of deaths (19.7%, 43) were caused by rodenticides from 1999 to 2008, with pesticides (37.6%) representing a significant cause of intoxication fatalities. Our data comparing 1999–2008 with 2009–2021 showed that the percentages of deaths due to organophosphorus (10.6 vs. 18.0%, χ^2^ = 4.902, *p* = 0.027); ethanol (10.1 vs. 18.0%, χ^2^ = 5.602, *p* = 0.018); amphetamine (0.0 vs. 4.6%, χ^2^ = 8.333, *p* = 0.004); and phosphorus rodenticides (0.0 vs. 4.6%, χ^2^ = 8.333, *p* = 0.004) were increased. However, the percentages of deaths due to tetramine (17.9 vs. 2.8%, χ^2^ = 26.823, *p* < 0.001), and carbon monoxide (16.5 vs. 8.3%, χ^2^ = 6.756, *p* = 0.009) were decreased. The distribution of intoxications due to the various toxic substances according to sex is shown in Table 4. Alcohol (*n* = 28, 21.2%); insecticides (*n* = 23, 17.4%); illicit drugs (*n* = 16, 12.1%); and other compounds (*n* = 16, 12.1%) caused fatal intoxications more in males, whereas insecticides (*n* = 24, 28.2%); alcohol (*n* = 14, 16.5%), and rodenticides (*n* = 12, 14.1%) involved more females (Table 4).

**Table 3:** Number of deaths by agent in the two periods (1999–2008 and 2009–2021) (4).

**Toxic agent**	**2009–2021**		**1999–2008**		***χ*** ^**2**^ **-test**	
	***n***	**%**	***n***	**%**	χ^2^	***p*** **-value**
Pesticide	72	33.2	82	37.6	0.935	0.333
Insecticides	47	21.7	35	16.1	2.304	0.129
Organophosphorus^*^	39	18.0	23	10.6	4.902	**0.027**
Others	8	3.7	12	5.5	0.819	0.365
Herbicides	5	2.3	4	1.8	-	0.994
Paraquat	4	1.8	0	0	2.285	0.131
Others	1	0.5	4	1.8	0.800	0.371
Rodenticide^*^	20	9.2	43	19.7	11.207	**0.001**
Phosphorus rodenticides^*^	10	4.6	0	0.0	8.333	**0.004**
Tetramine^*^	6	2.8	39	17.9	26.823	**< 0.001**
Organic fluoride rodenticides	2	0.9	3	1.4	–	1.000
Others	2	0.9	1	0.4	–	0.997
Alcohols^*^	42	19.4	27	12.4	3.958	**0.047**
Methanol	3	1.4	5	2.3	0.123	0.726
Ethanol^*^	39	18.0	22	10.1	5.602	**0.018**
Illicit drugs	20	9.2	13	6.0	1.642	0.200
Amphetamine^*^	10	4.6	0	0.0	8.333	**0.004**
Heroin	0	0.0	5	2.3	3.219	0.073
Morphine	4	1.8	4	1.8	–	1.000
Others	1	0.5	4	1.8	0.800	0.371
≥2 drugs	5	2.3	0	0.0	3.256	0.071
Prescription medicine	8	3.0	17	7.8	3.394	0.065
Sedative hypnosis	6	2.5	5	2.3	0.098	0.754
Anesthetics	1	0.0	7	3.2	3.160	0.075
Others	1	0.5	5	2.3	1.507	0.220
Other compounds	20	9.2	12	5.5	1.495	0.221
Succinylcholine	4	1.8	0	0.0	2.285	0.131
Cyanide	6	2.8	3	1.4	0.463	0.496
Nitrite	5	2.3	2	0.9	0.590	0.442
Others	5	2.3	7	3.2	0.333	0.564
Toxic animals and plants	16	7.4	16	7.3	–	0.989
CO^*^	18	8.3	36	16.5	6.756	**0.009**
Combined intoxication	11	5.1	5	2.3	2.307	0.129
Metal (salts)	6	2.8	6	2.8	–	1.000
Unknown	4	1.8	4	1.8	–	1.000
Total	217	100	218	100	–	–

χ^2^, Chi-squared; significant *p*-values are in bold (^*^p < 0.05).

**Table 4:** Sex distribution of intoxication deaths from 2009 to 2021.

**Toxic agents category**	**Male**		**Female**		χ^**2**^**-test**	
	***n***	**%**	***n***	**%**	χ^2^	***p*** **-value**
Rodenticide^*^	8	6.1	12	14.1	4.012	**0.045**
Insecticides	23	17.4	24	28.2	3.562	0.059
Herbicides	3	2.3	2	2.4	-	1.000
Alcohols	28	21.2	14	16.5	0.745	0.388
Drugs	16	12.1	4	4.7	3.398	0.065
Prescription medicine	1	0.8	7	8.2	6.173	0.013
Other compounds	16	12.1	4	4.7	3.398	0.644
Toxic animals and plants^*^	14	10.6	2	2.4	5.157	**0.023**
CO	9	6.8	9	10.6	0.966	0.326
Metal (salts)	3	2.3	3	3.5	0.016	0.899
Combined intoxication	8	6.1	3	3.5	0.263	0.608
Unknown	3	2.3	1	1.2	0.005	0.945

χ^2^, Chi-squared; significant *P*-values are in bold (^*^p < 0.05).

### 3.5. Manner of death

Among the 217 intoxication cases, 131 (60.4%), 57 (26.3%), 14 (6.5%), and 15 (6.9%) were accidental deaths, suicides, homicides, and uncertain, respectively. The manner of death reported based on the type of intoxications is presented in Table 5. The most common types of intoxications to which accidental deaths were attributable were alcohol (*n* = 42, 32.1%), illicit drugs and prescription medicines (*n* = 22, 16.8%), and toxic plants and animals (*n* = 16, 12.2%). Pesticides account for the largest proportion of intoxications deaths due to suicide (45, 78.9%). The extensive data analysis from 1999 to 2021 revealed a strong correlation between the cause and manner of death. Accidental intoxication deaths are usually unmotivated, while suicides and homicides are considered to be motivated. The number of cases where pesticides (*p* < 0.0001) were used in suicide and homicide cases was more than that in the accidental cases. On the contrary, accidents included accidental alcohol overdose (*p* < 0.0001), toxic animals and plants intoxication (*p* = 0.002), and combined intoxication (*p* = 0.023). All the results are summarized in Table 6.

**Table 5:** Statistics of toxic agents and manner of death reported from 2009 to 2021 at the Tongji Forensic Medicine Center in Hubei, China.

**Manner of death**	**Pesticide**	**CO**	**Illicit drugs and prescription medicine**	**Alcohols**	**Toxic animals and plants**	**Other compounds**	**Metal (salts)**	**Combined intoxication**	**Unknown**	**Total**
Accident	13	13	22	42	16	8	4	9	4	131
Suicide	45	3	4	0	0	4	0	1	0	57
Homicide	6	0	1	0	0	6	0	1	0	14
Unknown	8	2	1	0	0	2	2	0	0	15
Total	72	18	28	42	16	20	6	11	4	217

**Table 6:** The motivation for intoxication deaths in 1999–2021.

**Toxic agent**	**Suicide and homicide**		**Accident**		***p-value***
	***n***	**%**	***n***	**%**	
Pesticide^*^	51	71.8	13	9.9	**< 0.001**
Other compounds	10	14.1	8	6.1	0.057
Illicit drugs and prescription medicine	5	7.1	22	16.8	0.052
CO	3	4.2	13	9.9	0.197
Metal(salts)	0	0.0	4	3.1	0.338
Alcohols^*^	0	0.0	42	32.8	**< 0.001**
Toxic animals and plants^*^	0	0.0	16	12.2	**0.002**
Combined intoxications^*^	0	0.0	9	6.9	**0.023**
Unknown	0	0.0	4	3.1	0.338

χ^2^, Chi-squared; significant *p*-values are in bold (^*^p < 0.05).

## 4. Discussion

This study recorded 217 intoxication autopsy instances at TCMEH from 2009 to 2021. Comparing 2009–2021 and 1999–2008 showed that there were 4753 and 2416 autopsy cases, respectively (Table 1). Finally, intoxication deaths decreased from 9.0% in 1999–2008 (4) to 4.7% in 2009–2021. This decrease (*p* < 0.0001) may be attributable to a rise in autopsies and a decline in deaths from intoxication by some substances, especially carbon monoxide and tetramine, between 2009 and 2021.

### 4.1. Sex and age

Compared with our previous 1999–2008 report (4), by 2009–2021, alcohol (*n* = 28) had surpassed insecticides (*n* = 23) as the leading cause of intoxication-associated deaths in males. Rodenticides (*p* = 0.045) accounted for more deaths in females than in males. Toxic plants and animals (*p* = 0.023) accounted for a greater proportion of intoxication deaths among males than that among females (Table 4). Males tend to be more exposed to alcohol because of socialization and work pressure in China due to the traditional family structure where men work outside the home and women care for the family (7). Since men are more inclined to use ethanol because of these reasons, they are more susceptible to ethanol intoxications (8). Differences were also observed in the distribution of the manner of death among the sexes. Our research shows that men die from accidents at a higher rate than women (*p* = 0.005), whereas women are more likely to die by suicide than men (*p* = 0.006) (Table 7). In preventing female suicide, the issue of domestic violence cannot be disregarded. Family marriage conflicts in China frequently involve domestic violence, and more than 99% of those who engage in domestic violence during these conflicts are men (9). Therefore, it is essential to strengthen the protection of women's rights and interests, and legal aid agencies should intensify efforts to promote the law and educate and inspire women to use the legal system to their advantage bravely and effectively.

**Table 7:** Manner of death by sex in intoxication deaths from 2009 to 2021.

**Manner of death**	**Male**		**Female**		χ^**2**^**-test**	
	***n***	**%**	***n***	**%**	χ^2^	***P*** **-value**
Accident^*^	89	67.4	41	48.2	7.927	**0.005**
Suicide^*^	24	18.2	33	38.8	7.511	**0.006**
Homicide	10	7.6	4	4.7	0.706	0.401
Unknown	9	6.8	7	8.2	0.152	0.697

χ^2^, Chi-squared; significant *P*-values are in bold (^*^P < 0.05).

### 4.2. Toxic agents

#### 4.2.1. Pesticide intoxications

Similar to the Shenyang, China findings (10), pesticides led to 33.2% of all intoxication-related deaths during this study period. Overall, 47, 5, and 20 of the 72 deaths caused by pesticides were due to insecticide, herbicide, and rodenticide exposure, respectively. Most deaths were due to suicide (45, 62.5%), followed by accidents (13, 18.1%) and (6, 8.3%) homicides. Of the 39 cases of organophosphorus insecticide intoxications, 18 were due to oral administration of dichlorvos. The primary reason for this is the existence of numerous highly hazardous and relatively inexpensive organophosphorus insecticides worldwide (11).

The World Health Organization (WHO) estimates that there are approximately one million cases of pesticide intoxications annually, resulting in ~20,000 deaths globally (12). According to a nationally representative survey of suicide mortality in India, pesticides are commonly used as suicide tools (13), with the majority using insecticides, as in China. Since 2001 (except for 2005), the decline in their pesticide suicide rate has accelerated (14). China's agricultural industry has outlawed the production and sale of highly dangerous pesticides, such as methomyl. However, since some pesticides are still on the market, the number of pesticide suicides has not decreased significantly.

In the latest study, paraquat exposure resulted in four deaths, unlike that reported in the prior study. Additionally, two of the cases involved murder. There have been similar complaints in other regions of China (15). In one of our cases, the suspect applied it to the deceased's undergarments by applying a small quantity each time. As a result, the clinical onset of the sickness was sluggish, and it was difficult to identify the components of paraquat in the body at the late stage due to the body's metabolism; this, together with the concealed methods of the crime, made it challenging to solve such murder. Since July 2014, China has revoked the registration and manufacturing license for liquid paraquat, and has permitted production only for export. The domestic sale and usage were discontinued in July 2016, and its soluble gel has been prohibited since September 2020 (16).

Compared to that in 1999–2008 (4), the proportion of deaths due to rodenticide intoxications was substantially reduced in the present study (9.2% in 2009–2021 vs. 19.7% in 1999–2008, χ^2^ =11.207, *p* = 0.001), mainly owing to the decline in tetramine intoxications (2.8% in 2009–2021 vs. 17.9% in 1999–2008, χ^2^ =26.823, *p* < 0.001). In 2013, China's Ministry of Agriculture demanded a “designated area” for selling highly dangerous pesticides and acquiring pesticides with recognizable brand names. Simultaneously, the government seized and cleared up tetramine and strictly enforced the policy to avoid harm from tetramine (17).

However, the proportion of phosphine rodenticides has increased (from 0.0% in 1999–2008 to 4.6% in 2009–2021, χ^2^ = 10.282, *p* = 0.001). Phosphate intoxication is the fourth most prevalent rodenticide intoxication in the United States, whereas aluminum phosphide is widely used in developing nations (18). Notably, 10 of the 20 deaths attributed to rodenticide intoxications in this study were the result of unintentional phosphine inhalation. Aluminum phosphide and zinc phosphide react with water in the air and hydrochloric acid in the stomach to produce phosphine gas, which is highly poisonous (19–21). According to our research, phosphine was frequently associated with household intoxication whereby the deceased belonged to four distinct families, each with more than two victims. These accidental fatalities were the result of indoor use and inappropriate storage of solid phosphides. Our research also showed that children had a lower tolerance for phosphine inhalation than adults, and 9 of the 10 deaths occurred in children under 13 years old, with the youngest being 7 months old. According to previous studies, many phosphine intoxication victims are children (22), and children are more sensitive to phosphine intoxication (23). Even when all intoxication victims are children, the younger the victim, the more severe the intoxication symptoms. Improving the packaging of phosphine rodenticides and keeping them out of the reach of children would prevent accidental exposure to children. The government and schools should also enhance pesticide safety education for children.

#### 4.2.2. Alcohol

We observed 39 deaths (25 males and 14 females) due to ethanol intoxications. The proportion of ethanol intoxication deaths significantly increased from 10.1% in 1999–2008 (4) to 18.0% in 2009–2021 (*p* = 0.018), and this may be related to the rising socialization and life demands. Conroy and Visser reported that not drinking alcohol is construed as strange behavior (24). Most ethanol-related fatalities occurred in males (64.1%), and the youngest victim was only 17 years old. WHO's 2018 Global Status Report on Alcohol and Health reveals that more than 3 million people die annually due to alcohol, with more than three-quarters of those deaths occurring in men (25).

The acetaldehyde dehydrogenase gene mutation rate in the Asian population is higher than that in the European and American populations (26). Therefore, Asians are more susceptible to severe intoxications and even death. According to the fifth edition of forensic toxicology, the blood concentration of ethanol intoxication is 100 mg/dL, whereas the lethal blood concentration is 400–500 mg/dL (5). Given the identification of ethanol production by postmortem microbial action, a blood ethanol/n-propanol concentration ratio >20 indicates that the individual consumed alcohol during their lifetime. The average blood ethanol content of those who died from ethanol intoxication was 434.06 ± 133.5 mg/dL (range: 199.1–843.5 mg/dL). Our data show that among the 39 cases of death by ethanol intoxications, 21 had ethanol blood concentrations of 400 mg/dL or more; In the remaining 18 cases, the concentration of ethanol in the blood exceeded the level for severe intoxication. We examined these 18 decedents and discovered that they had coronary heart disease, cardiomyopathy, chronic alcoholism, and alcoholic coma, respectively, but ethanol intoxication was their leading cause of death. A decedent with severe fatty infiltration of theatrio-ventricular node who died after consuming alcohol in our practice had a measured blood ethanol concentration of 199.1 mg/mL, indicating a large individual variation. Furthermore, we observed variations in the blood ethanol concentrations among the deceased, which might be related to the time between death and the toxicological test as well as individual differences (27).

#### 4.2.3. Illicit drugs and prescription medicines

In China, illicit drug-related cases are mainly handled by public security, in which we have less. Twenty overdose cases involving illicit drugs, including narcotics (heroin and morphine) and psychoactive substances (methamphetamine and ketamine), have been identified. There were 10 deaths caused by amphetamine overdose, an emerging phenomenon, in 2009–2021 (including combined intoxication with amphetamines and other illicit drugs; χ^2^ = 8.333, *p* = 0.004) at a mean age of 37 ± 8.8 years. Amphetamines, synthetic, addictive, mood-altering drugs, are used illegally as a stimulant (28). According to WHO, illicit drug misuse is a major concern among high school students (29). Notably, no deceased individuals were reported to have used more than two medicines concurrently between 1999 and 2008; nevertheless, five cases were discovered in our most recent investigation. This indicates that, despite China's rigorous anti-drug policies, the targeted substances are still being obtained illegally. The average age of individuals who died from drugs intoxication was 36.3 (range: 23–66) years, and 89.5% were aged 20–49 years. The synergistic effects of various drugs and the assessment of their lethal doses are of particular importance to forensic toxicologists, and as such, these issues necessitate not only objective toxicology reports but also a reliance on the empirical judgment of forensic scientists.

There were eight prescription medication-related deaths, seven involving sedative-hypnotic medications, such as phenothiazine, clozapine, diphenhydramine, and amitriptyline, and one involving to insulin overdose *via* injection. The psychological tolerance of individuals declines gradually as society develops, and the incidence of drug intoxications rises due to various reasons including family, society, survival pressure, and emotional stress. From 1997 to 2003, a retrospective analysis of acute intoxication cases at the First Hospital of China Medical University's emergency department revealed that sedative-hypnotic medications accounted for 30.3% of medication intoxication, which warrants further study (30).

#### 4.2.4. Other compounds and metal salts

In this category, five of six cyanide- and five succinylcholine-related deaths were linked to animal hunting. These two chemicals are frequently used to produce “poison darts” for illegal animal hunting but can also be used for murder. The government should increase patrols and early warning systems to combat the unlawful sale of slingshots and poisoned darts and to tighten down on illegal hunting and sale of wildlife.

Three of the victims were aged under 10 (7 months, 2, and 6 years), making all four nitrite intoxication deaths food-related. This shows that children are more likely to mistakenly consume foods with high nitrite levels than adults and that children have a higher mortality risk from intoxications. This may be due to body weight and food intake; as children consume more food per unit of body weight than adults, making them more vulnerable to nitrite intoxication. To ensure food cleanliness and safety, the appropriate departments should improve food oversight while increasing public awareness of proper food handling and preservation techniques.

#### 4.2.5. Toxic plants and animals

In the present study, 16 deaths due to toxic animals or plants included 10 cases caused by aconitine intoxications, two strychnine intoxications, two brucine intoxications, and two from snakebites. According to the previous study's findings, aconitine intoxications remained the leading cause of death in this category (4). All 10 cases were due to improper use, also consistent with the previous report (31). Aconite is included in traditional Chinese medicine, and its improper or un-constituted form, overdose, and misuse of that meant for external use as for internal drink are the common causes of intoxications (32). To prevent such accidents, we recommend that the Market Supervision Administration and other relevant departments strengthen the supervision and management of the production, processing, and use of toxic herbal medicines, and prohibit the private production of medicinal wine for consumption and sale. Additionally, we encourage the general population to receive herbal medications from formal Chinese hospitals rather than private clinics.

#### 4.2.6. CO

From 2009 to 2021, 18 cases of CO intoxications occurred, and the average concentration of carboxyhemoglobin (HbCO) in the blood was 61.3 ± 12.0% (39–83.4%). Compared to the 1999 to 2008 data, the number of deaths due to CO intoxication has decreased significantly (36 cases, 16.5%) from our institution (*p* = 0.009). One of the following three conditions can easily lead to CO intoxications in China: gas water heaters in the bathroom are the most common source of CO intoxications, followed by burning coal for warmth while sleeping and sleeping with the windows closed or in a vehicle while using air conditioning. Natural gas and solar energy have increasingly replaced gas water heaters in several northern regions, resulting in a gradual decrease in CO intoxications.

Deaths caused by CO intoxication were mostly accidental, consistent with reports from northeastern China (33). When individuals keep their windows closed in the winter to block out the cold, they are more likely to accidentally poison themselves with CO. As an example, we had a situation where a father left his 5-year-old son in the passenger seat of a small car and got out of the car without turning the car off or opening the windows for about 40 min, after which the youngster had no indications of life. All parents should remember that leaving their children in a car is never a good idea, and if they must, they should at least make sure the car is turned off and the windows are cracked open for air circulation before leaving. We urge market supervision departments to tightly regulate the manufacturing and sale of gas stoves and gas water heaters and not to overlook CO in vehicles. Centralized heating areas may effectively lower the chances of intoxication due to CO, and their expansion is what we are advocating for.

#### 4.2.7. Combined intoxication

In total, 11 accidental deaths occurred due to combined intoxication from different agents. Four groups of combined intoxication were found: CO and alcohol (1 case), CO and illicit drugs (2 cases), alcohol and prescription medications (2 cases), and alcohol and illicit drugs (6 cases). Lee showed that illicit drug users with concomitant alcohol abuse have a significantly higher mortality rate than the general population (34). According to previous reports (31), death from CO combined with illicit drug intoxication may be because illicit drugs increase the metabolic rate and oxygen consumption of the body, thus, exacerbating the death process of CO intoxications. Therefore, recognizing that illicit drugs, alcohol, and CO predispose to increased death susceptibility, especially potentially preventable deaths, could help develop preventive measures.

### 4.3. Manner of death

According to Table 5, the most common type of poison used in suicides was pesticides (45 cases, 78.9%). A better understanding of the emotional dynamics of suicidal patients is possible by implementing a community psychological counseling and security system. Several studies have linked suicidal ideation and behavior to the availability of lethal means (35), and strict control of suicide tools will effectively reduce the suicide rate. In homicide cases, “other compounds” (succinylcholine, *n* = 4; concentrated sulfuric acid, *n* = 1; and cyanide, *n* = 1) and pesticides (*n* = 6) were the most common.

The manner of death can reflect the motivation for intoxication. Pesticides are more likely to be blamed for motivated deaths, like suicide and homicide. Deaths caused by alcohol, CO, illicit drugs, prescription medicines, and toxic animals and plants are always accidental. As concluded by Moebus and Bödeker, reducing the acquisition of pesticides has a positive effect on reducing the incidence of pesticide intoxications (12). A study of a community cluster randomized trial of household pesticide lock-up storage in rural Asia found that locking up pesticides did not reduce suicide mortality (36). Therefore, only banning highly toxic pesticides at the source of pesticide production is effective in reducing deaths from suicide *via* pesticide administration. Regarding CO and alcohol, safety education should be further developed, with a focus on appropriate use. For prescription medicine, it is important to emphasize following medical advice and not overdosing, as well as with the use of herbal medicines; we advocate going to a regular Chinese hospital to obtain them. Lastly, the government should take severe measures against illicit drug-related criminal activities and enhance the status and function of human intelligence in anti-drug efforts.

Regarding limitations, the data used were from a forensic pathology laboratory, where intoxication-related deaths are investigated, and it excludes cases from the whole of China. However, this retrospective analysis may reflect the intoxication situation in central China to a certain extent. It provides valuable information for medical examiners and policemen when handling such intoxication cases and useful suggestions for improving public safety policies.

## 5. Conclusion

Compared to the 1999–2008 period, the percentage of intoxication-related deaths evaluated at TCMEH dropped compared to that in the 2009–2021 period. Effective tetramine control and increased safety standards for residential gas use in China are likely responsible for the notable decline in the percentage of rodenticide and CO intoxication cases. Insecticides are the leading cause of death, followed by alcohol and illicit drugs. We propose strengthening the supervision of organophosphorus pesticides and pesticide regulatory departments within their scope of responsibility to effectively supervise and manage pesticide production, transportation, and sales, among other issues. Increases in alcoholic intoxication fatalities have been observed. Furthermore, amphetamine-related deaths have emerged in recent years, and the use of multiple illicit drugs has increased. This suggests that although the government is taking severe measures against the transportation and distribution of illicit drugs, the current anti-drug intelligence mechanism may be imperfect. The emergence of some “non-contact” illicit drugs poses new challenges for anti-drug efforts, requiring the development of a sound and sustainable system. Forensic professionals face new challenges because of evolving intoxication trends that result in death, such as the toxicology of combination intoxication, methods for detecting novel poisons, and identifying some intoxication-related homicide cases that go undetected. Our retrospective research included two deaths from snake bites, which is interesting to note. Forensic professionals should be aware of any minor skin lesions while conducting post-mortem examinations.

## Data availability statement

The original contributions presented in the study are included in the article/supplementary material, further inquiries can be directed to the corresponding author.

## Ethics statement

Written informed consent was obtained from the individual(s), and minor(s)' legal guardian/next of kin, for the publication of any potentially identifiable images or data included in this article.

## Author contributions

LLih and WY searched for available studies and completed the manuscript. LLia made pictures and tables. JM, LX, HH, LLia, and LZ provided assistance for the whole research process. LQ designed the study, guided the writing of the paper, and made revisions. All authors have read and agreed to the published version of the manuscript.
